# Influence of Initial Gap, Voltage, and Additives on Zinc Microcolumn Morphology by Local Electrochemical Deposition

**DOI:** 10.3390/s25020521

**Published:** 2025-01-17

**Authors:** Yi Liu, Fuliang Wang

**Affiliations:** 1 State Key Laboratory of High Performance Complex Manufacturing, Changsha 410083, China; 193701010@csu.edu.cn; 2 School of Mechanical and Electrical Engineering, Central South University, Changsha 410083, China

**Keywords:** local electrochemical deposition, zinc microcolumns, applications of bioabsorbable metals, glucose chemical sensors

## Abstract

Local electrochemical deposition (LECD) is an innovative additive manufacturing technology capable of achieving precise deposition of metallic microstructures. This study delves into the ramifications of pivotal operational parameters—namely, the initial electrode gap, deposition voltage, and additive concentration—on the morphology of zinc microcolumns fabricated through LECD. A holistic approach integrating experimental methodologies with finite element simulations was adopted to scrutinize the influence of these variables on the microcolumns’ dimensions, surface morphology, and structural integrity. The findings reveal that augmenting the initial electrode gap results in microcolumns with larger diameters. Conversely, the deposition voltage primarily modulates the formation rate without exerting a notable impact on the columns’ dimensional attributes. The incorporation of additives enhances surface smoothness and diminishes column diameters; however, an overabundance of additives adversely affects the overall microstructure. Optimal parameters for the production of high-quality zinc microcolumns were determined to be a deposition voltage of 3.4 V and an electrode gap of 10 μm. These discoveries contribute pivotal insights for the refinement of LECD processes, with particular relevance to biomedical applications, such as the development of zinc-based bioabsorbable materials for orthopedic implants and cardiovascular devices.

## 1. Introduction

Local electrochemical deposition (LECD) has emerged as a novel additive manufacturing methodology, grounded in the precision of electroforming and electroplating processes. In this technique, metal ions are directed to a specified area through the meticulous control of an electric field, resulting in their deposition. A notable advantage of LECD lies in its high deposition rate, which facilitates the creation of intricate structures of various shapes and heights onto the surfaces of metals, conductive polymers, and semiconductors, regardless of their underlying geometries and thicknesses. This versatility ensures a broad spectrum of applicability for LECD. The concept of LECD was first introduced by Madden et al. in 1995, who demonstrated its potential by successfully fabricating zinc columns and spiral springs using a Watts plating solution [1,2]. These pioneering studies underscored the feasibility of micro-manufacturing through LECD. However, the initial deposited structures exhibited rough surfaces and inconsistent diameters. In response, numerous researchers have delved into enhancing the surface quality of these deposits by meticulously controlling parameters such as the anode motion [3,4,5,6], solution composition [7], anode size, and fabrication techniques [8,9,10], as well as exploring new application domains [11]. Through rigorous investigation, the effects of these various parameters have been elucidated, paving the way for the successful fabrication of both two-dimensional and three-dimensional structures using LECD [12].

Zinc-based biomaterials have recently attracted significant attention as a novel category of bioabsorbable metallic materials, particularly in medical applications such as orthopedics and cardiovascular stenting. Compared to other degradable metallic biomaterials, such as magnesium- or iron-based options, zinc biomaterials display a more balanced corrosion rate and notably do not produce hydrogen gas. Animal studies have shown that zinc biomaterials elicit minimal to moderate toxicity and immune responses, which are comparable to those seen with AZ31 magnesium alloys. Notably, there have been no reports of extensive cell death or foreign body reactions. As a result, zinc biomaterials have emerged as a promising candidate for bioabsorbable metallic applications in cardiovascular and orthopedic fields. Furthermore, zinc oxide and zinc, due to their low cost and high sensitivity, exhibit favorable catalytic activity, biocompatibility, and environmental benignity, making them suitable for the fabrication of glucose chemical sensors [13,14]. Given this potential, the deposition of zinc micro- and nano-structures is of particular importance. Wang Chunyao et al. have made contributions to this area by depositing copper–zinc alloy and pure zinc micropillars, and they have investigated the influence of different initial gaps on the alloy composition [15,16]. Their findings offer valuable insights for optimizing zinc-based biomaterial deposition processes [17].

Currently, the research pertaining to micro-manufacturing utilizing liquid electrochemical deposition (LECD) remains scant. Furthermore, the literature concerning the manufacture of zinc micropillars is similarly sparse. Notably, the existing corpus of research on the fabrication of zinc micropillars is equally constrained. Importantly, a comprehensive theoretical framework that elucidates the regulatory mechanisms through which diverse parameters and additives influence the microstructure morphology has yet to be formulated. Specifically, there is an urgent need for thorough investigations to discern the mass-transfer processes and the underlying physical phenomena associated with the electrochemical reactions that occur during the sedimentation phase [18,19].

## 2. Experiment

### 2.1. Experimental Equipment

**Figure 1:**
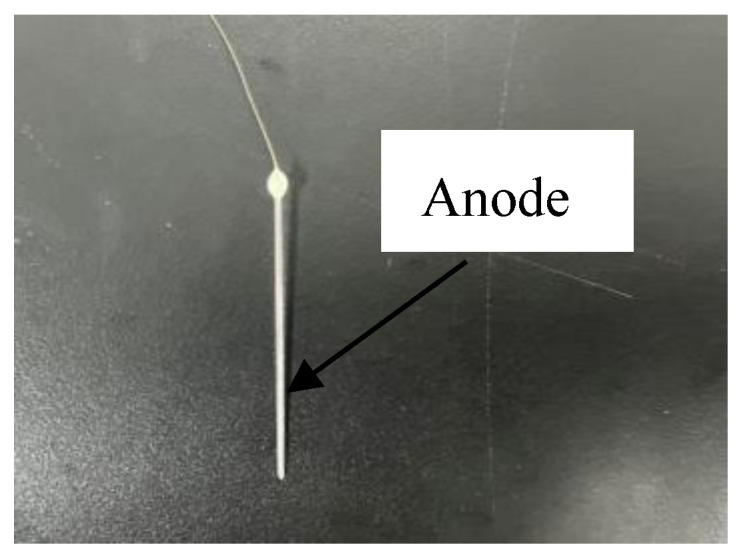
Photograph of the prepared anode needle.

**Figure 2:**
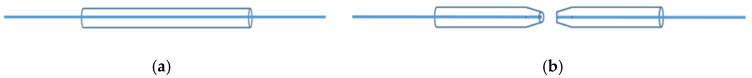
Schematic of the anode fabrication process. (**a**): The glass tube with the metal wire. (**b**): Two needles were formed by pulling with the pull needle instrument.

The accuracy of local electrochemical deposition should reach the micron level, and the anode cannot be dissolved in the solution. Therefore, a platinum wire with a diameter of 20 μm was selected as the conductive material of the anode. A 2~3 cm tin wire with a diameter of 20 μm and a long copper wire with a length of 5 cm were welded together at one end, and then this welded metal wire is placed in the center of a quartz glass tube (inner diameter: 0.25 mm). The glass tube with the metal wire was placed in a needle puller, and two needles were formed by pulling. Finally, the two needles were polished by a precision needle grinder (EG-401, NARISHIGE Co., Ltd., Tokyo, Japan). The prepared anode needle is shown in Figure 1, while Figure 2a,b depicts a schematic of the anode fabrication process.

**Figure 3:**
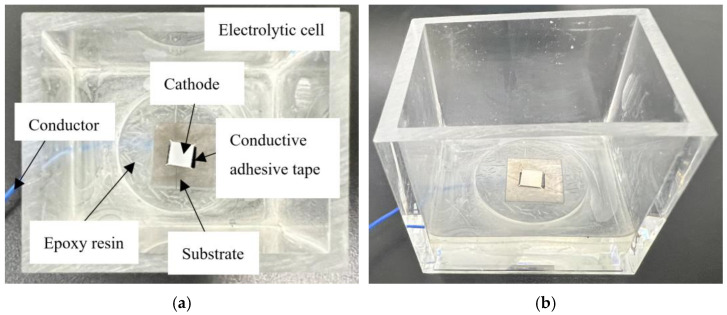
Photographs of the prepared cathode electrolytic cell. (**a**):The top-view representation of the prepared cathode electrolytic cell. (**b**): the side-view illustration of the prepared cathode electrolytic cell.

Initially, a tungsten sheet with dimensions of 15 × 15 × 5 mm and a wire were securely bonded using a conductive adhesive. During the experimental procedure, the wire was subsequently connected to the negative electrode of the external power source. Subsequently, the tungsten sheet, along with a segment of the wire, was encapsulated within epoxy resin. This encapsulation step served to electrically isolate the junction between the tungsten sheet and the wire from the electrolyte. Once the resin had thoroughly cured, the exposed surface of the tungsten sheet underwent preparation, which involved grinding it with sandpaper and polishing it with an alumina polishing agent to attain a smooth and glossy finish. Finally, the fabricated cathode was firmly mounted at the base of an acrylic tank, marking the completion of the cathode tank’s construction. The resultant cathode cell configuration is depicted in Figure 3a,b.

To facilitate the production of 3D microstructures, a motion platform equipped with at least three degrees of freedom was deemed essential. Consequently, a platform capable of translating along the *x*-, *y*-, and *z*-axes was constructed utilizing three linear translation stages (M-L01, Physik Instrumente Ltd., Shanghai, China). This setup was governed by a motor controller, ensuring motion accuracy of 0.05 μm, which adequately met the experimental requirements. In the course of the deposition process, the anode underwent upward movement, with a velocity of 3000 μm/s.

### 2.2. Experimental Solution

In this study, four experimental solution formulations were employed: the original solution, devoid of any additives, and three additional solutions, designated as solutions 1–3, containing varying concentrations of additives. The additives utilized were Atotech zinc 290A (Cl) carrier additive and Atotech zinc 290MIX supplement agent, sourced from the Atotech Company (Berlin, Germany). The detailed compositions of these solutions are outlined in Table 1.

The solutions were prepared by a systematic process involving several steps. Initially, 30 g/L of zinc chloride (ZnCl_2_) and 250 g/L of potassium chloride (KCl) were dissolved in hot water (60 °C). Simultaneously, 30 g/L of boric acid (H_3_BO_3_) was dissolved in hot water (80 °C). These two solutions were then mixed at a 4:1 ratio. Subsequently, 1–2 mL/L of hydrogen peroxide and 1–2 g/L of zinc powder were added to the mixture, which was thoroughly stirred for 2 h to ensure the replacement of other heavy metal ions. The resultant solution was filtered to eliminate any precipitates. For solutions 1–3, the additives—Atotech zinc 290A (Cl) carrier additive and Atotech zinc 290MIX supplement agent—were diluted 5–8 times with water and gradually introduced into the solution under continuous stirring. The pH values of the solutions were subsequently adjusted to fall within the range of 5.5 to 6.2 using 5% hydrochloric acid and 5% sodium hydroxide.

The post-treatment procedure following the zinc deposition encompassed several steps. Initially, the zinc deposits were rinsed thoroughly with distilled water to ensure the removal of residual contaminants. Subsequently, the samples were subjected to cold air drying to eliminate the moisture content. Upon the successful completion of this treatment process, the specimens were meticulously observed and characterized using precise methodologies to evaluate their physical and functional properties, as well as their overall performance.

### 2.3. Electrode Reactions

The original solutions comprised ZnCl_2_, KCl, and H_3_BO_3_.

The electrochemical behavior of ZnCl_2_ in solution can be described by the equation ZnCl_2_ + H_2_O. During hydrolysis, the reaction of ZnCl_2_ + H_2_O ⇌ Zn(OH)Cl + HCl occurs, generating heat and enhancing the turbidity of the water. To mitigate this, weakly acidic solutions were employed to induce a homo-ionic effect, thereby suppressing the hydrolysis reaction. Additionally, Zn ions have the potential to combine with OH ions to form Zn(OH)_2_ deposits prior to their deposition as metallic Zn through the reaction Zn^2+^ + 2OH^−^ ⇌ Zn (OH)_2_. The use of weakly acidic solutions prevented this reaction by minimizing the concentration of free hydroxide ions. In such solutions, ZnCl_2_ ionizes into Zn^2+^ and Cl^−^ ions, as represented by the equation ZnCl_2_ ⇌ Zn^2+^ + 2Cl^−^.

KCl is a robust electrolyte that undergoes complete ionization into K^+^ and Cl^−^ ions in water, denoted by the equation KCl ⇌ K^+^ + Cl^−^. KCl enhanced the current efficiency of the solution and, among various galvanizing processes, it exhibited the highest current efficiency, significantly improving the coverage capability of the solution.

To generate a weakly acidic solution environment, H_3_BO_3_ (boric acid) was employed through a series of chemical reactions.

B(OH)_3_ + 2H_2_O ⇌ [H_3_O_3_-B ← OH]^−^ + H_3_O^+^ and B(OH)_3_ + H_2_O ⇌ (H_4_BO_4_)^−^ + H^+^

The electrochemical reactions occurring at the cathode involved the reduction of Zn^2+^ ions to Zn metal, denoted by Zn^2+^ + 2e^−^ → Zn, and the reduction of protons to hydrogen gas, denoted by 2H^+^ + 2e^−^ → H_2_↑. Conversely, at the anode, the oxidation of hydroxyl ions led to the evolution of oxygen gas and the formation of water, as represented by 4OH^−^ − 4e^−^ → O_2_↑ + 2H_2_O. Consequently, during the course of these reactions, Zn was deposited onto the cathode, while H_2_ and O_2_ were generated at their respective electrodes.

The carrier additive was employed as a grain-refining agent, whereas the supplement agent served as a grain-leveling agent.

## 3. Experimental Results and Discussion

### 3.1. Effects of the Initial Gap and Voltage on Zinc Deposition

**Figure 4:**
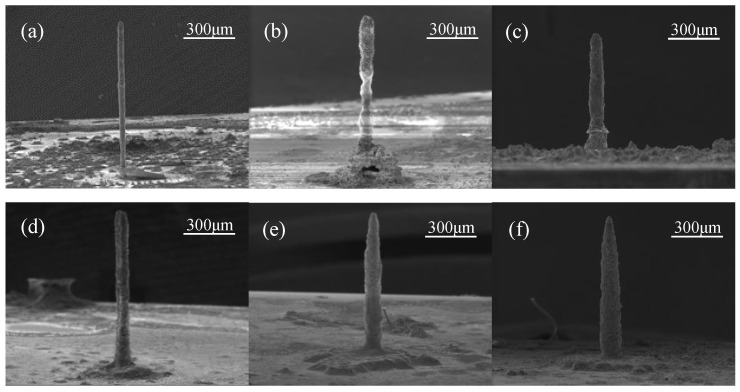
SEM images showing the morphology of the zinc microcolumns produced via LECD with a deposition voltage of 4.0 V and initial gaps of (**a**) 4 μm, (**b**) 8 μm, (**c**) 12 μm, (**d**) 16 μm, (**e**) 20 μm, and (**f**) 24 μm.

LECD experiments were conducted utilizing the original solution, with an applied voltage of 4.0 V and an initial electrode gap ranging from 4 to 24 μm. The SEM (scanning electron microscope) images of the deposited zinc columns are presented in Figure 4, while the corresponding diameters of these columns are tabulated in Table 2.

Under the influence of the electric field, zinc ions progressively precipitated onto the cathode plate. As the initial gap between the electrodes widened, the diameter of the deposited zinc columns increased accordingly. This correlation can be attributed to the gradual decrease in electric field intensity distribution with an increasing gap, which led to slower deposition and consumption rates of zinc ions at the reaction center. Consequently, zinc ions had sufficient time to diffuse from high to low concentration regions within the solution, ensuring the timely replenishment of ions consumed across the entire reaction area. This resulted in the formation of increasingly larger zinc columns, with a gradual transition in shape from cylindrical to tower-like. As the deposition gap further increased, the electric field strength decreased, allowing sediments at the edges to grow well. Consequently, redundant sediments formed around the base of the zinc columns.

The deposition voltage was varied in the experimental setup. Specifically, when the applied current was less than 0.2 A, no precipitation of zinc occurred to form discernible columns. Upon increasing the current density of the electric field, a notable augmentation was observed in the rate of column formation. Nonetheless, the deposition voltage exhibited no statistically significant influence on the diameters of the resultant columns.

### 3.2. Effects of the Additives on the Structure and Surface Morphology of the Zinc Columns

**Figure 5:**
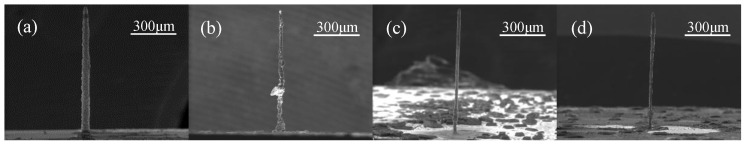
SEM images showing the morphology of the zinc microcolumns produced via LECD with a deposition voltage of 4.0 V, an initial gap of 5 μm, and (**a**) the original solution, (**b**) solution 1, (**c**) solution 2, and (**d**) solution 3. (The compositions of the solutions are given in Table 1).

**Figure 6:**
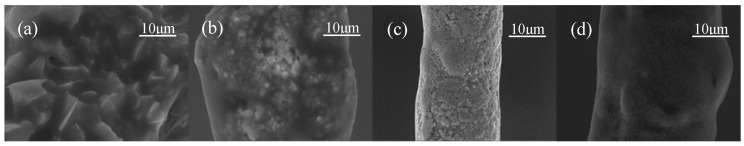
SEM images showing the surface morphology of the zinc microcolumns produced via LECD with a deposition voltage of 4.0 V, an initial gap of 5 μm, and (**a**) the original solution, (**b**) solution 1, (**c**) solution 2, and (**d**) solution 3. (The compositions of the solutions are given in Table 1).

The addition of additives at appropriate concentrations reduced the diameter and improved the surface morphology of the deposited columns. As demonstrated in Table 3, there was a decrease in the diameters of the Zn columns with an increase in the additive concentration, up to a specified threshold. Nevertheless, beyond this threshold, an excessive amount of additive adversely impacted the shape and surface morphology of the columns, resulting in an augmentation of their diameter. SEM images of the zinc columns, fabricated via LECD using various solutions, a voltage of 4.0 V, and an initial gap of 5 μm, are presented in Figure 5 and Figure 6.

As an organic additive, the grain refiner exhibited selective adsorption or reduction in the region of high current density on the cathode. This process effectively inhibited the discharge of metal complex ions or the surface diffusion of adsorbed metal atoms. Consequently, the overpotential of the cathode reaction was elevated, which resulted in a deceleration of the electrode reaction rate, thereby obtaining a fine and smooth coating. Consequently, numerous additives have demonstrated remarkable efficacy as grain refiners.

Chemical adsorption entails the creation of chemical bonds and the selective attachment of additives onto the cathode surface, where these additives undergo coordination reactions with metal ions present in the solution. The resultant species formed on the metal surfaces are termed surface complexes. In the present investigation, these surface complexes were found to impede the discharge of metal ions, thereby significantly elevating the reaction’s overpotential. This condition favored the formation of novel crystal nuclei, ultimately leading to the precipitation of relatively smaller sediment grains. Concurrently, the diameter of the deposited columnar material was diminished due to the influence of the current density distribution during the additive adsorption reaction, which enhanced the localized nature of the deposition. The procured additives, comprising multiple components, also exhibited a physical adsorption effect. This physical adsorption primarily arose from van der Waals forces acting between the additive and the metal electrode. Given the relatively weak nature of these forces, the additives were prone to detachment, exerting a comparatively minor influence on the overpotential.

**Figure 7:**
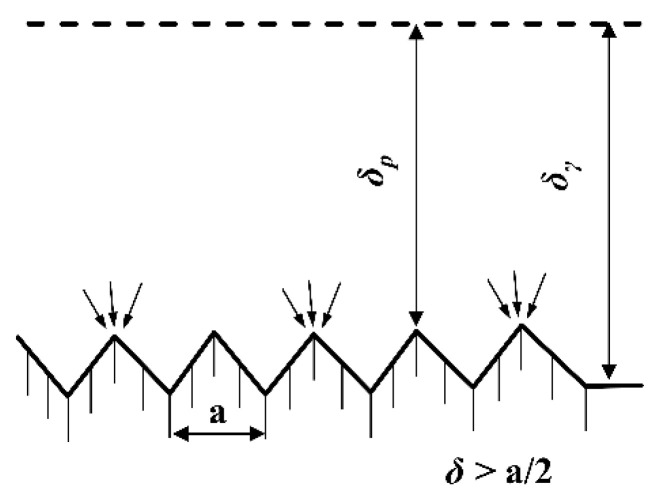
Diffusion layers on microscopic surfaces.

The supplement agent is synonymous with a crystal grain leveling agent. On microscopic concave and convex surfaces, the effective diffusion layer at the valleys (δ_γ_) exhibited a greater thickness compared to that at the peaks (δ_p_), as illustrated in Figure 7. Consequently, the diffusion of the leveling agent occurred more rapidly at the peaks than at the valleys, resulting in a higher concentration at the peaks relative to the valleys. This concentration gradient led to a more pronounced inhibitory effect of the leveling agent on the deposition reaction at the peaks. In contrast, the deposition rate was enhanced at the valleys, giving rise to the leveling effect. Consequently, the surface of the sedimentary column attained a smooth and delicate finish [20].

When the concentration of the leveling agent was excessively low, it failed to accumulate adequately at either the peaks or valleys, resulting in the absence of an inhibitory effect, and consequently, no leveling effect was observed. Conversely, when the concentration of the leveling agent was excessively high, it accumulated at both the peaks and valleys, exerting an inhibitory effect across the entire surface and thereby failing to produce a leveling effect. Therefore, the leveling effect was solely observed when the concentration of the leveling agent was maintained at an optimal level.

## 4. Simulation of the LECD Manufacturing Process for Zinc Microcolumns

Finite element simulations were employed to model LECD experiments, with the aim of analyzing the electric field distribution, elucidating the influences of additives, and gaining insights into the material transfer processes and electrochemical reactions occurring during the deposition phase. COMSOL Multiphysics^®^ 6.2, a comprehensive finite element simulation software developed by COMSOL (Sweden), was utilized for this purpose, given its extensive application across numerous professional domains.

The local electrochemical deposition (LECD) process fundamentally involves a chemical reaction that occurs when the electrode potential deviates from its equilibrium state due to the application of a voltage differential between the anode and cathode. In the absence of an applied voltage across these electrodes, the oxidation reaction at the anode is equivalent to the reduction reaction at the cathode, resulting in a balanced state of charge transfer and mass transfer throughout the system. Consequently, no current flows through the electrodes during this equilibrium condition.

Upon initiating LECD, the application of a bias voltage induces a current flow within the circuit. This current facilitates the reduction of local metal cations as they migrate toward the cathode surface. Consequently, the concentration of ions within the electroplating solution evolves over time. Therefore, the kinetics of this process can be described using the Butler–Volmer equation:

(1) $$i_{0}=i^{0}\left[\exp\left(\frac{\alpha nF}{RT}\eta_{c}\right)-\frac{c_{Zn^{2+}}}{c_{bulk}}\exp\left(-\frac{\beta nF}{RT}\eta_{c}\right)\right],$$

where *i*^0^ denotes the exchange current density, *α* represents the charge transfer coefficient in the positive direction, and *β* signifies the charge transfer coefficient in the negative direction. The symbol *n* stands for the number of electrons involved in the reaction, *R* is the universal gas constant, *T* indicates the ambient temperature, and *F* is the Faraday constant. Furthermore, $c_{{Zn}^{2+}}$ represents the concentration of zinc ions, while $c_{bulk}$ denotes the bulk concentration of ions in the solution. Additionally, *η_c_* is the overpotential, which is defined as

(2) $$\eta_{c}=\phi_{s}-\phi_{l}-E_{eq},$$

where *ϕ_s_* and *ϕ_l_* represent the potentials of the cathode and the electrolyte, respectively, and *E_eq_* denotes the equilibrium potential.

During the reduction of zinc ions, the geometric configuration of the system undergoes continuous alterations, and the boundary conditions at the cathode are accordingly defined as

(3) $$N_{Zn^{2+}}\cdot \vec{n}=\frac{-i_{loc}}{2F}$$

where $N_{{Zn}^{2+}}$ signifies the number of zinc ions, $\vec{n}$ denotes the direction normal to the cathode boundary, and *i_loc_* represents the current density during the restoration process.

In the proposed model, all boundaries are assumed to be in an insulated and flux-free state, with the exception of the micro-anode and cathode boundaries. Consequently,

(4) $$N_{i}\cdot \vec{n}=0,$$

where *N_i_* is the flux of each ion species.

From the law of conservation of materials, we can infer that

(5) $$\frac{\partial c_{i}}{\partial_{t}}+\nabla \cdot N_{i}=0,$$

where *c_i_* is the concentration of zinc ions in the electrolyte.

According to Faraday’s law, the rate of deposition of zinc ions is

(6) $$v_{c}=\frac{M_{Zn}i_{loc}}{2\rho_{Zn}F},$$

where *M_Zn_* and *ρ_Zn_* are the molar mass and density of zinc, respectively.

Owing to the electronegativity of the plating solution throughout the LECD process,

(7) $$\sum z_{i}c_{i}=0,$$

where *z_i_* is the number of charged zinc ions. Moreover, the electron mobility *u_m,j_* is

(8) $$u_{m,j}=\frac{D_{i}}{RT}$$

where *D_i_* is the zinc ion diffusion coefficient.

The solution maintained electrical neutrality when a platinum wire served as the micro-anode and a tungsten sheet acted as the cathode. Given the close proximity of the ion concentrations and material characteristic coefficients, the primary parameters were established as outlined in Table 4.

## 5. Simulation Results and Analysis

**Figure 8:**
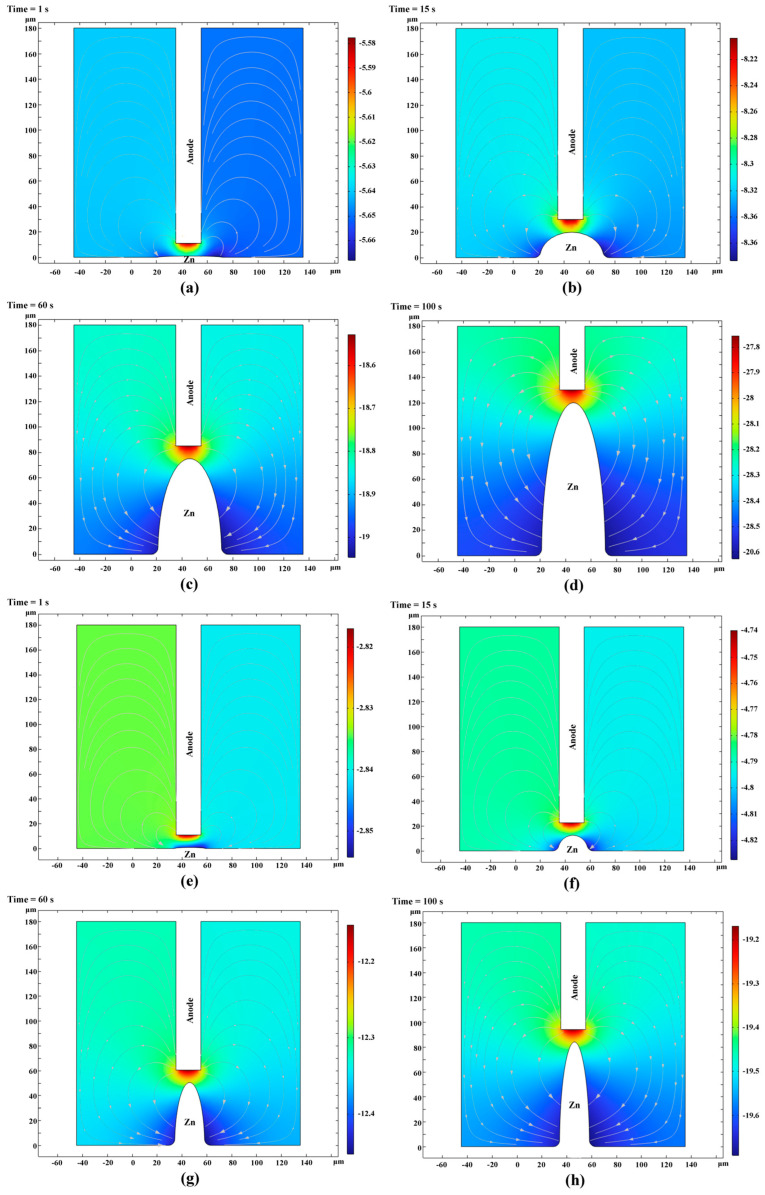
Potential cloud in the solution at different points in time during the deposition process. (Streamlines: Electrolyte current density vector line: ABS (cd. itot) (A/m^2^); surface: electrolyte potential (V)) ((**a**–**d**) and (**e**–**h**) depict the potential contours and concentration fields at various time points during the deposition process for electrolytes using the base solution and the solution supplemented with additives, respectively).

The growth of the zinc microcolumns was analyzed through simulation. Figure 8a–d and Figure 8e–h depict the potential contours and concentration fields at various time points during the deposition process for electrolytes using the base solution and the solution supplemented with additives, respectively. The zinc microcolumns at different stages exhibited similar concentration fields and potential distribution patterns. High concentrations and high potentials were concentrated between the sediment apex and the anode. As the distance from this region increased, both the concentration and potential decreased, with the left and right sides displaying symmetrical characteristics.

**Figure 9:**
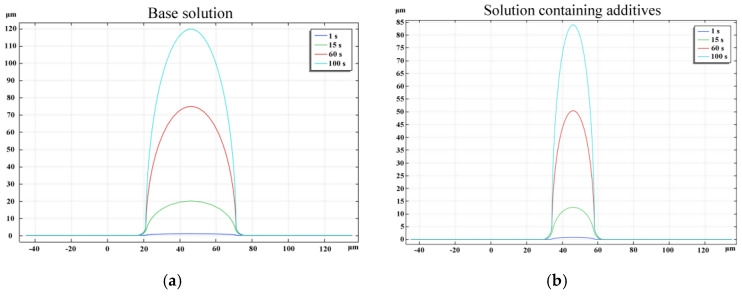
Deposition morphology of zinc at different times.

Figure 9a illustrates the geometric contour morphology of the zinc microcolumns produced using the base solution at various stages during the deposition process. After 1, 15, 60, and 100 s, the microcolumns attained heights of 1.2, 18, 72, and 120 µm, respectively. Due to the symmetrical distribution of the electrolyte potential and concentration field, the deposition of zinc ions exhibited a high degree of symmetry. The diameters remained relatively consistent across different time points, with only a slight diffusion arc observed at the base of the column. This suggests a predominantly vertical growth pattern. This phenomenon arose because, although the zinc ions underwent adsorption and reduction across the entire surface of the column, the highest potential and concentration values were localized near the anode, leading to a faster deposition rate in this region. Consequently, the diameter of the microcylinder remained largely unchanged over time.

Figure 9b depicts the geometric contour morphology of the zinc microcolumns formed using a solution containing additives (Solution 2) at various stages during the deposition process. The microcolumns attained heights of 0.9, 13.5, 54, and 90 μm. A comparison between Figure 9a,b reveals that the diameter of the microcolumns was smaller when the additive-containing solution was utilized, aligning with the experimental findings.

## 6. Conclusions

This study employed a combination of practical experiments and mathematical simulations to investigate the impact of various parameters on zinc microcolumns produced via LECD. The following conclusions were derived from the results:

1. As the initial gap size increased, the diameter of the sedimentary column expanded. Concurrently, an increase in the deposition voltage led to an augmentation in the current density of the deposition electric field, which in turn accelerated the rate of columnar formation.
2. Carrier additives and photoagents, at appropriate concentrations, effectively refined the surface morphology and reduced the diameter of the microcolumns. However, an excessive content of these additives adversely affected the shape, surface morphology, and diameter of the columns.
3. The experimental results indicate that a deposition voltage of 3.4 V and a longitudinal spacing of 10 μm yielded the optimal deposition efficiency and surface topography.

The deposited microcolumns exhibit versatility in potential applications, including the production of hydrophobic surfaces with self-cleaning capabilities, enzyme-free glucose chemical sensors [21], and zinc microwire implants for promoting bone integration, conduction, and/or induction in organisms. Consequently, they offer a wide array of potential uses across diverse fields.

## Figures and Tables

**Table 1:** Electrodeposition solution composition and working conditions.

Solution	ZnCl_2_ Content [g/L]	KCl Content [g/L]	H_3_BO_3_ Content [g/L]	pH	Carrier Additive Volume [mL/L]	Supplement Agent Volume [mL/L]
Original	30	250	30	5.5–6.2	-	-
Solution 1	30	250	30	5.5–6.2	5	0.125
Solution 2	30	250	30	5.5–6.2	2.5	0.0625
Solution 3	30	250	30	5.5–6.2	1.25	0.03125

**Table 2:** Relationship between sedimentary gap and sedimentary column diameter.

Sedimentary gap [μm]	4	8	12	16	20	24
Sedimentary column diameter [μm]	30	68.7	75.17	80	107.75	115.89

**Table 3:** Relationship between the additive content and the column diameter.

Solution	Original Solution	Solution 1	Solution 2	Solution 3
Sedimentary column diameter [μm]	50	26	24	20

**Table 4:** The main parameters used for the simulation.

Symbol	Numerical Value	Physical Meaning
*T*	323 K	Temperature
*i* ^0^	9.393 A/m^2^	Exchange current density
*Eeq_rel*	0.11 V	Balanced potential
*alpha_c*	0.31	Cathode transfer coefficient
*alpha_a*	0.31	Anode transfer coefficient
*phis_anode*	4.0 V	Potential
*z_c1*	2	Number of zinc ion charges
*D_c1*	1 × 10^−9^ m^2^/s	Zinc ion diffusion coefficient
*sigma*	0.1 S/m	Solution conductivity

## Data Availability

Data are contained within the article.
